# *Cnidium monnieri* Polysaccharides Exhibit Inhibitory Effect on Airborne Transmission of Influenza A Virus

**DOI:** 10.3390/v18010086

**Published:** 2026-01-08

**Authors:** Heng Wang, Yifei Jin, Yanrui Li, Yan Wang, Yixin Zhao, Shuang Cheng, Zhenyue Li, Mengxi Yan, Zitong Yang, Xiaolong Chen, Yan Zhang, Zhixin Yang, Zhongyi Wang, Kun Liu, Ligong Chen

**Affiliations:** 1 College of Veterinary Medicine, Hebei Agricultural University, Baoding 071000, China; wangheng946@163.com (H.W.); sh08120922@163.com (S.C.); lzy19971204@163.com (Z.L.); ymx76327806@163.com (M.Y.); yang5241225@163.com (Z.Y.); chen1571767@163.com (X.C.); 2 Academy of Military Medical Sciences, Academy of Military Sciences, Beijing 100071, China; christina_jyf@foxmail.com (Y.J.); lyr98@foxmail.com (Y.L.); w1377876707y@foxmail.com (Y.W.); yixinzhao1129@foxmail.com (Y.Z.); zany1983@163.com (Y.Z.); yy_xiao@126.com (Z.Y.); zhongyi_wang@foxmail.com (Z.W.)

**Keywords:** *Cnidium monnieri* polysaccharides, influenza A virus, airborne transmission, immunomodulatory effect, NF-κB pathway

## Abstract

Influenza A virus (IAV) continues to present a threat to public health, highlighting the need for safe and multi-target antivirals. In this study, anti-influenza activity, airborne transmission blocking capacity, and immunomodulatory effects of *Cnidium monnieri* polysaccharides (CMP) were evaluated. Cytotoxicity in A549 cells was assessed by CCK-8 (CC_50_ = 8.49 mg/mL), antiviral efficacy against A/California/04/2009 (CA04) by dose–response (EC_50_ = 1.63 mg/mL), and the stage of action by time-of-addition assays (pre-, co-, post-treatment). A guinea pig model infected with CA04 was used for testing the effect of pre-exposure CMP on transmission, with readouts including nasal-wash titers, seroconversion, lung index, and tissue titers (EID_50_). RT-qPCR was employed to quantify the mRNA expression levels of proinflammatory cytokines, including TNF-α, IL-1β, and IL-6, in lung tissue, while Western blot analysis was performed to assess the expression and phosphorylation status of key proteins involved in the NF-κB signaling pathway. CMP suppressed viral replication in vitro within non-cytotoxic ranges, and pre-treatment—rather than co- or post-treatment—significantly reduced titers and cytopathic effect, consistent with effects at pre-entry steps and/or host priming. In vivo, pre-exposure CMP lowered nasal shedding, reduced aerosol transmission (3/6 seroconverted vs. 6/6 controls), decreased lung indices, and diminished tissue viral loads; IAV was undetectable in trachea at 7 days post-infection in pre-exposed animals, and nasal-turbinate titers declined relative to infection controls. Moreover, during in vivo treatment in mice, CMP significantly suppressed the levels of inflammatory cytokines (TNF-α, IL-1β, and IL-6) in lung tissue. This effect was mechanistically associated with CMP-mediated regulation of the NF-κB signaling pathway, leading to attenuation of inflammatory responses. These data indicate that CMP combines a favorable in vitro safety and efficacy profile with inhibition of airborne spread in vivo, supporting further mechanistic, pharmacokinetic, and fractionation studies toward translational development.

## 1. Introduction

Influenza A virus (IAV) infects a wide range of hosts and can cross species barriers among birds, mammals, and humans [1,2]. It remains a persistent threat to global public health and causes substantial economic losses to the livestock and poultry industries [3,4,5]. Vaccines and antiviral drugs are the mainstays of prevention and treatment [6,7]. However, vaccine effectiveness depends heavily on accurate strain prediction, while existing antiviral drugs frequently encounter issues of resistance and adverse effects [8,9,10]. Therefore, developing broad-spectrum, low-resistance, and safe antiviral agents remains an urgent necessity [11,12,13]. Natural medicines have attracted increasing attention owing to their multi-target mechanisms and low toxicity [14,15,16]. Among them, Traditional Chinese Medicines (TCM) comprise a large repertoire of bioactive constituents, including polysaccharides, flavonoids, and alkaloids, many of which exhibit antiviral effects against influenza viruses through both direct inhibition and immunomodulatory pathways [17,18].

Excessive cytokine release—cytokine storms and viral pneumonia—often constitute major causes of respiratory injury and mortality in severe influenza patients [19]. Accordingly, modulating cytokine expression is a critical therapeutic strategy for influenza virus infection [20]. The inflammatory response triggered by viral infection relies on the activation and transduction of multiple signaling pathways, with the NF-κB signaling pathway serving as a key mediator in regulating immune and inflammatory responses [21]. As the core transcription factor of the classical NF-κB signaling pathway, p65 serves as a key regulatory protein in cellular responses to pathogen invasion [22]. Under resting conditions, p65 typically binds to the inhibitory protein IκBα and exists in an inactive form within the cytoplasm. When pathogens are recognized by cellular pattern recognition receptors, they activate the upstream IKK complex. IKK subsequently phosphorylates IκBα, leading to its ubiquitination and degradation, thereby releasing p65. p65 activation depends not only on dissociation from IκBα but critically on its own phosphorylation at specific serine residues. Phosphorylated, activated p65 (p-p65) rapidly translocates to the nucleus, binds to κB sites on DNA, and initiates transcription of downstream proinflammatory cytokines (e.g., TNF-α, IL-6), chemokines, and interferons [23]. Accumulating evidence indicates that activation of the NF-κB signaling pathway exacerbates IAV-induced lung injury [14]. Therefore, therapeutic modulation of NF-κB signaling to attenuate inflammatory cytokine production is of particular importance for the development of novel anti-influenza agents.

Aerosol and droplet transmission play a central role in the rapid spread of IAV among humans and animals [24,25]. Fine respiratory particles (<5 µm) exhaled by infected hosts can remain airborne for extended periods, facilitating long-distance dissemination and interspecies transmission [26]. Viral stability and infectivity are strongly influenced by environmental conditions such as temperature and relative humidity, and even short-range aerosol exposure can establish productive infection in mammalian models [27,28]. Airborne IAV has been detected in clinical and agricultural environments, emphasizing the bioaerosol-associated risk of outbreaks in both humans and livestock [29]. Thus, interventions capable of reducing viral shedding and interrupting airborne transmission are of high public-health and veterinary importance [30,31].

*Cnidium monnieri* (*C. monnieri*) Cusson (“Shechuangzi”) is a traditional herbal medicine containing abundant coumarins, volatile oils, chromones, flavonoids, and trace elements [32]. Recent pharmacological studies have highlighted its antiviral potential—compound formulations of *C. monnieri* show synergistic therapeutic effects against viral infections, and its signature coumarins display direct antiviral activities, including inhibition of HSV-1, suppression of HBV secretion, and blockade of HIV-1 Rev nuclear export [33,34,35]. Moreover, *C. monnieri* demonstrates bronchodilatory and anti-asthmatic properties that may help alleviate influenza-induced respiratory symptoms [36]. Its polysaccharides, the major macromolecular components, have been reported to enhance macrophage activation, humoral immune responses, and T-cell proliferation via upregulating IL-2 and IFN-γ secretion [37,38,39]. However, the anti-influenza potential of *C. monnieri* polysaccharides (CMP) remains largely unexplored. Here, we evaluated the safety profile, anti-influenza efficacy, capacity to block airborne transmission, and immunomodulatory effects of CMP against influenza A virus.

## 2. Materials and Methods

### 2.1. Viruses and Cells

Influenza A virus A/California/04/2009 (H1N1) was provided by the Institute of Microbiology, Chinese Academy of Sciences [40], and amplified in 9-day-old specific pathogen-free (SPF) eggs (Beijing Boehringer Ingelheim Viton Biotechnology Co., Ltd. Beijing, China). The harvested whole-capsid fluid was then inoculated into SPF chicken eggs to determine the egg infectious dose 50% (EID_50_). The titer of the virus stock solution was determined by the chicken embryo titration method to be 10^7.45^ EID_50_/mL. A549 cells (CCL-185, ATCC) were cultured in Dulbecco’s Modified Eagle Medium (DMEM, Gibco, Grand Island, NY, USA) supplemented with 10% fetal bovine serum (FBS, Gibco, Grand Island, NY, USA) and 100 U/mL penicillin–streptomycin (P/S) under conditions of 37 °C and 5% CO_2_. H1N1 virus (A/California/04/2009 (CA04)) was serially diluted 10-fold in DMEM medium containing 2% FBS and 2% dual antibiotics. Each dilution was inoculated into a 96-well plate containing A549 cells at 0.1 mL/well. After incubation at 37 °C for 1 h, the incubation supernatant was discarded, and A549 cell maintenance medium (100 μL/well) was added. After 72 h of incubation at 37 °C in a cell culture incubator, the hemagglutination activity of the cell supernatants in each well was measured according to the WHO hemagglutination test guidelines. The number of agglutinations for each concentration gradient was recorded, and the tissue culture infectious dose 50% (TCID_50_) of the virus was calculated. EID_50_ and TCID_50_ values were calculated using the Reed–Muench method.

### 2.2. Maximum Non-Toxic Concentration in A549 Cells

CMP were purchased from Moqi Biotechnology Co., Ltd. (Shanghai, China). Detailed information on monosaccharide composition, molecular weight distribution, and sample purity is provided in the Supplementary Materials. A solution containing 2% fetal bovine serum (FBS) and 1% (*w*/*v*) penicillin–streptomycin in DMEM medium was prepared. A two-fold serial dilution method was used to create a series of solutions at different concentrations (10.125 mg/mL, 6.75 mg/mL, 4.5 mg/mL, 3 mg/mL, 2 mg/mL, 1.33 mg/mL). A549 cells were revived, passaged 3–5 times to stabilize growth, harvested, and seeded uniformly into 96-well plates in DMEM growth medium containing 10% FBS and 2% (*w*/*v*) penicillin–streptomycin. After 24–48 h at 37 °C and 5% CO_2_, when monolayers reached ~80% confluence, medium was removed, cells were washed twice with PBS containing 2% penicillin–streptomycin, and CMP dilutions were added (100 µL/well; five replicates per concentration). Cell-only (vehicle) and blank (medium-only) controls were included [41].

Cell viability was determined using the CCK-8 assay kit (Beyotime Biotechnology, Shanghai, China) [42]. After 72 h infection, wells were washed twice with PBS (2% penicillin–streptomycin), 100 µL fresh medium was added, and 10 µL CCK-8 reagent was added per well per the manufacturer’s instructions. Plates were incubated for 3 h, and absorbance at 450 nm was recorded. Cell viability was calculated as:

Cell Viability% = [(As − Ab)/(Ac − Ab)] × 100%

As = absorbance of the sample well (cells + culture medium + CCK-8 + test compound); Ac = absorbance of the cell control well (cells + culture medium + CCK-8; no test compound); Ab = absorbance of the blank well (culture medium + CCK-8; no cells, no test compound).

The maximum non-toxic concentration was determined from the concentration–viability relationship.

### 2.3. In Vitro Antiviral Efficacy and EC_50_ Assay

Starting from the CC_50_ of CMP in A549 cells, CMP was each two-fold serially diluted in DMEM containing 2% FBS. For infection, A549 cells (prepared as in Section 2.2) were washed twice with PBS (2% penicillin–streptomycin) and inoculated with 100 TCID_50_ of H1N1 (100 µL/well). After 1 h adsorption at 37 °C, inoculum was removed and serial drug dilutions were added (five replicates per concentration). Following 72 h incubation, cell viability was determined by CCK-8 (Section 2.2), and the EC_50_ of CMP was calculated from dose–response curves [43].

### 2.4. Time-of-Addition (Drug-Timing) Assay

A549 cells were seeded into 96-well plates and cultured to ~90% confluence. The concentration of CMP solution was 1.63 mg/mL. Dextran T-10 (NDC) was purchased from Solarbio Science & Technology Co., Ltd. (Shanghai, China). It was diluted to a concentration of 200 μg/mL using the same DMEM diluent. Oseltamivir (OS) was purchased from yuanye Bio-Technology Co., Ltd. (Shanghai, China) and prepared at a concentration of 100 μg/mL using the same DMEM diluent [44]. Different experimental groups were established:

(i) Pre-treatment (Pre Cell): Add CMP solution, 100 µL/well, and incubate at 37 °C, 5% CO_2_ for 2 h. The supernatant was removed, and cells were inoculated with diluted virus (100 TCID_50_; 100 µL/well) for 1 h at 37 °C. Inoculum was discarded and replaced with drug-free DMEM containing 2% FBS (DMEM–2% FBS), followed by incubation to 72 h.
(ii) Co-treatment: CMP and virus inoculum were mixed 1:1 (50 µL + 50 µL) and added to cells for 1 h at 37 °C, 5% CO_2_. The mixture was then replaced with drug-free DMEM–2% FBS and incubated to 72 h.
(iii) Post-treatment: Cells were first inoculated with virus (100 TCID_50_; 100 µL/well) for 1 h at 37 °C Remove the viral supernatant and cover with DMEM–2% FBS. After 2 h, remove the supernatant, DMEM–2% FBS containing CMP was added and cells were incubated to 72 h.

Culture supernatants were collected at 12, 24, 36, 48, 60, and 72 h. Viral titers were determined as TCID_50_ by the Reed–Muench method (as described in Section 2.1). A negative drug control group administered NDC (NDC) at 2 h post-infection and a positive drug control group administered OS (OS) at 2 h post-infection were established. Lesion development in each group was observed at 36 h post-infection.

### 2.5. Airborne-Transmission Experiment

To assess the impact of CMP on airborne transmission of influenza virus, guinea pigs were evaluated in CMP-treated and untreated control cohorts. The Hedley strain guinea pigs (body weight 300 g–350 g) used in this study were purchased from Vital River Laboratory Animal Technology Co., Ltd. (Beijing, China). All guinea pigs tested negative for IAV antibodies prior to the start of the experiment.

Thirty-six animals were randomized 1:1 to CA04 infection and CMP administration 2 h before infection groups (n = 18 per group) [45,46]. The treated group received CMP by oral gavage 2 h before inoculation (100 µL, 5 mg/mL); the control group received an equivalent volume of PBS 2 h before inoculation. All animals were anesthetized with isoflurane and intranasally inoculated with 100 µL of CA04 virus (10^6.0^ EID_50_). Animals were then allocated to three cages per group (6 per cage).

At 24 h post-inoculation, an additional 36 healthy guinea pigs were introduced (those corresponding to the treated cohort received CMP 2 h prior to infection, while those in the control cohort received an equal volume of PBS at the same time point). Three transmission models were established per group (n = 6 each): (i) direct-contact transmission, achieved by co-housing with inoculated animals; (ii) aerosol transmission, by housing in an adjacent cage separated by 5 cm (no physical contact); and (iii) indirect-contact transmission, by placing recipients into cages containing used bedding from inoculated animals. Nasal-wash samples were collected from both inoculated donors and recipients at 1, 3, 5, and 7 days post-infection (dpi) [47].

For sampling, animals were anesthetized with isoflurane. One milliliter of PBS containing penicillin–streptomycin was instilled into one nostril, and the effluent was collected from the contralateral nostril as the nasal wash. Samples were labeled and stored at −80 °C. To minimize cross-contamination, recipient animals were sampled first and inoculated donors last [48].

Viral titers (EID_50_) in nasal washes were determined in SPF embryonated chicken eggs. Frozen samples stored at −80 °C were thawed at 4 °C and subjected to 10-fold serial dilution in PBS containing antibiotics, with vortex mixing at each step. Diluted samples were inoculated into 9–10-day-old SPF eggs (200 µL/egg; 3 eggs per dilution) and incubated at 37 °C. Embryos dying nonspecifically within 24 h were excluded; remaining eggs were harvested at 72 h, and hemagglutination (HA) was assessed. EID_50_ values were calculated by the Reed–Muench method.

### 2.6. Hemagglutination-Inhibition (HI) Assay

At 21 dpi, sera were collected from all animals for HI testing following the OIE Manual of Diagnostic Tests and Vaccines for Terrestrial Animals [49].

### 2.7. Virus Tissue Distribution and Lung Index

Guinea pigs were randomized into two groups (n = 12/group): CA04 infection and CMP administration 2 h before infection groups. The procedure is the same as in 2.5. Both groups inoculated with CA04 virus (10^6.0^ EID_50_)., At 1, 3, 5, and 7 dpi, three animals per group were euthanized. Lung, nasal turbinates, and trachea were collected, homogenized in 1 mL PBS (tissue homogenizer; QIAGEN, Hilden, Germany), and clarified at 12,000× *g*, 4 °C, 10 min [50]. Supernatants were stored at −80 °C and titrated for EID_50_ in eggs as above. An additional 24 guinea pigs were used to establish a blank control group receiving only CMP and a blank control group receiving only PBS, with 12 animals in each group. Lung index (lung weight/body weight) was determined on the same days to assess disease severity.

### 2.8. Effects of CMP on Cytokine-Related mRNAs in Virus-Infected Mouse Lungs

The experiment utilized 140 female Balb/c mice weighing 18–20 g, purchased from Vital River Laboratory Animal Technology Co., Ltd. (Beijing, China). During the study period, mice were housed in an IVC system and provided with standard chow and water. The 140 mice were randomly divided into 7 groups of 20 mice each: negative control group (NC), virus-infected group (PC), negative drug treatment group (NDC), oseltamivir treatment group (OS), low-dose *Cnidium monnieri* polysaccharide treatment group (CMP-L), medium-dose *Cnidium monnieri* polysaccharide treatment group (CMP-M), and high-dose *Cnidium monnieri* polysaccharide treatment group (CMP-H). Mice in all groups except the NC group were lightly anesthetized with isoflurane and intranasally inoculated with 50 μL of H1N1 subtype influenza A virus (CA04, 10^6.0^ EID_50_) per mouse. Mice in the NC group received intranasal inoculation with PBS at the same dose. All groups of mice received treatment starting on the day of infection, 2 h after establishing the influenza virus-infected mouse model, with continuous treatment for 5 days. Treatment regimens were as follows: CMP-L, CMP-M, and CMP-H groups received CMP at concentrations of 2 mg/mL, 5 mg/mL, and 10 mg/mL, respectively, at 50 μL per mouse; OS group received OS treatment at a dose of 20 mg/kg, diluted in phosphate-buffered saline (PBS) at a concentration of 5 mg/mL [51]; NDC group received NDC administration. NDC was diluted to a concentration of 10 mg/mL in phosphate-buffered saline (PBS) and administered at a dose of 50 μL per animal.

NC group and PC group mice were administered PBS at 100 μL per animal. All treatments were administered via oral gavage once daily at the same time each day. Five mice were randomly selected from each group at 1, 3, 5, and 7 dpi, euthanized by decapitation, the collected mouse lung tissue was divided into two portions. The first portion was homogenized and subjected to viral load determination (EID_50_), calculated using the Reed–Muench method. The second portion was used for RNA extraction and subsequent measurement of cytokine-related mRNA expression levels. Total RNA was extracted from mouse lung tissue using the RNeasy Plus Universal Kit (73404, Qiagen, Germany). Reverse transcription was performed using the Prime Script™ RT Kit (RR047A, Takara, Dalian, China). Reverse transcription-quantitative polymerase chain reaction (RT-qPCR) was performed using Power SYBR™ Green PCR Master Mix. RT-qPCR was conducted on an ABI 7500 Fast Real-Time PCR System (Thermo Fisher Scientific, Waltham, MA, USA). The RT-qPCR program consisted of 50 °C for 2 min, 95 °C for 10 min, 40 cycles: 95 °C for 15 s, 60 °C for 1 min. Data analysis employed the 2^−ΔΔCt^ method. PCR primers synthesized by Sangon Biotech Co., Ltd. (Shanghai, China). Sequences as follows: TNF-α forward, 5′-CCCTCACACTCAGATCATCTTCT-3′; TNF-α reverse, 5′-GCTACGACGTGGGCTACAG-3′; IL-1β forward, 5′-GCAACTGTTCCTGAACTCAACT-3′; IL-1β reverse, 5′-ATCTTTTGGGGTCCGTCAACT-3′; IL-6 forward, 5′-TAGTCCTTCCTACCCCAATTTCC-3′; IL-6 reverse, 5′-TTGGTCCTTAGCCACTCCTTC-3′; GAPDH forward, 5′-AGGTCGGTGTGAACGGATTTG-3′; GAPDH reverse, 5′-TGTAGACCATGTAGTTGAGGTCA-3′.

### 2.9. Regulatory Effects of CMP on NF-κB p65 and p-NF-κB p65 Protein Expression in Mouse Lung Tissue

A total of 35 female Balb/c mice were randomly divided into 7 groups of 5 mice each. Following the grouping method described in Section 2.8, they were randomly divided into 7 groups of 5 mice each, following the grouping method described in Section 2.8. An influenza virus-infected mouse model was established via intranasal inoculation, using the same inoculation method as in Section 2.8. Two hours after establishing the influenza virus-infected mouse model, mice in each group began treatment on the day of infection, continuing for 5 consecutive days. Treatment and administration methods were identical to those in Section 2.8. At 5 dpi, five mice from each group were sacrificed. Lung tissue was collected from these mice and lysed using RIPA buffer (Epizyme, Shanghai, China) to extract total protein from mouse lung tissue. Protein concentration was measured using the BCA Protein Quantification Kit (Beyotime Biotechnology, Shanghai, China). Equal amounts of protein were subjected to SDS-PAGE electrophoresis and transferred to PVDF membranes (Merck Millipore, Burlington, MA, USA). Subsequently, membranes were incubated overnight with mouse monoclonal antibodies: NF-κB p65 (1:3000, Proteintech, Manchester, IL, USA), phosphorylated NF-κB p65 (1:5000, Proteintech, Manchester, USA), and GAPDH (1:8000, Proteintech, Manchester, USA), followed by detection with HRP-conjugated goat anti-rabbit or anti-mouse IgG. Bands were visualized by Clarity Western ECL Substrate (Bio-Rad, Hercules, CA, USA) in a luminescent image analyzer. The protein expression levels were quantified using ImageJ software (version 1.54g; National Institutes of Health, Bethesda, MD, USA) and normalized with GAPDH. The level of protein phosphorylation was determined as the ratio of gray values for phosphorylated protein to total protein.

### 2.10. Statistical Analysis

Data are presented as mean ± SD. Analyses were performed using GraphPad Prism 10.5.0 (GraphPad Prism software, Inc San Diego, CA, USA). ANOVA with appropriate post hoc multiple-comparisons tests was used for continuous data, and viral titers were log10-transformed when applicable. Proportions (e.g., transmission/seroconversion rates) were compared using Fisher’s exact test. All tests were two-tailed; *p* < 0.05 was considered significant.

## 3. Results

### 3.1. Cytotoxicity and Antiviral Efficacy of CMP

**Figure 1:**
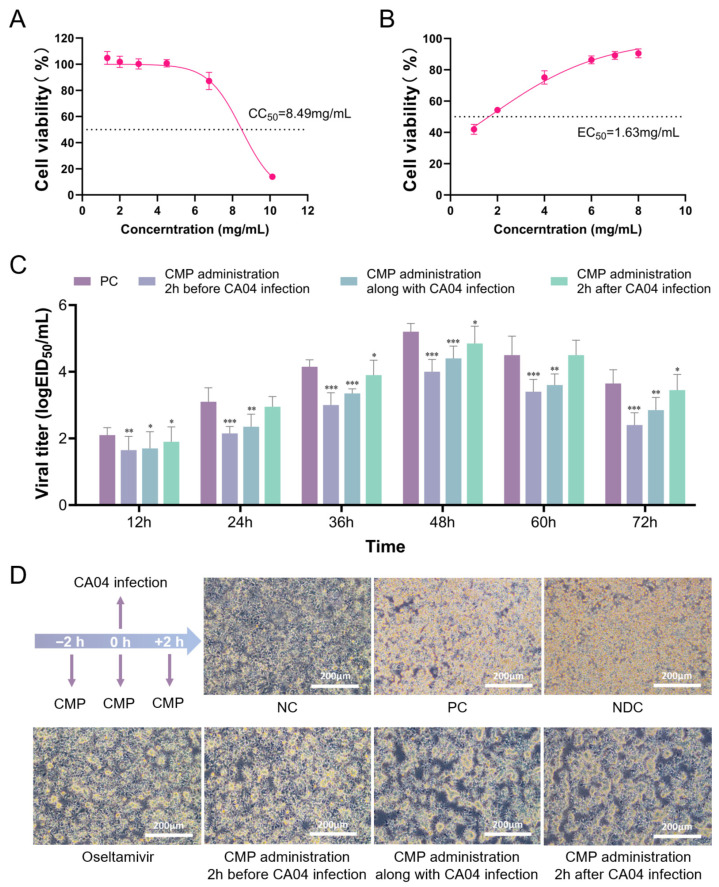
In vitro anti-influenza activity of CMP: (**A**) Cytotoxicity of CMP in A549 cells determined by the CCK-8 assay; (**B**) Antiviral efficacy of CMP within the non-cytotoxic concentration range assessed by CCK-8 following influenza virus infection; (**C**) Antiviral activity of CMP under three different treatment conditions: (i) Pre-treatment; (ii) Co-treatment; (iii) Post-treatment; * *p* < 0.05, ** *p* < 0.01, *** *p* < 0.001. (**D**) Microscopic observation of CPE changes under different treatment conditions 36 h post-infection. The scale bar represents 200 μm.

CMP was evaluated for cytotoxicity in A549 cells using the CCK-8 assay across graded concentrations to define a safe working range and an effective anti-influenza dose. CMP exhibited a CC_50_ of 8.49 mg/mL (95% CI: 8.25–8.73 mg/mL) in A549 cells (Figure 1A). Within the non-cytotoxic range, treatment of H1N1 (CA04)–infected A549 cells with CMP suppressed viral replication in a concentration-dependent manner, yielding an EC_50_ of 1.63 mg/mL (95% CI: 1.43–1.82 mg/mL) (Figure 1B).

### 3.2. Time-of-Addition Assay

Experimental results demonstrated that pre-treatment (CMP administration 2 h before CA04 infection) significantly reduced viral titers at all examined time points (Figure 1C). At 36 h post-infection, co-treatment (CMP administration along with CA04 infection) and post-treatment (CMP administration 2 h after CA04 infection) also significantly decreased viral titers, with pre-treatment showing the most pronounced inhibitory effect. These findings suggest that CMP may exert antiviral activity during the early phase of infection; however, further mechanistic studies are needed to determine the specific stage of the viral life cycle targeted by CMP.

### 3.3. CMP Reduces Aerosol Transmissibility of Influenza Virus

**Figure 2:**
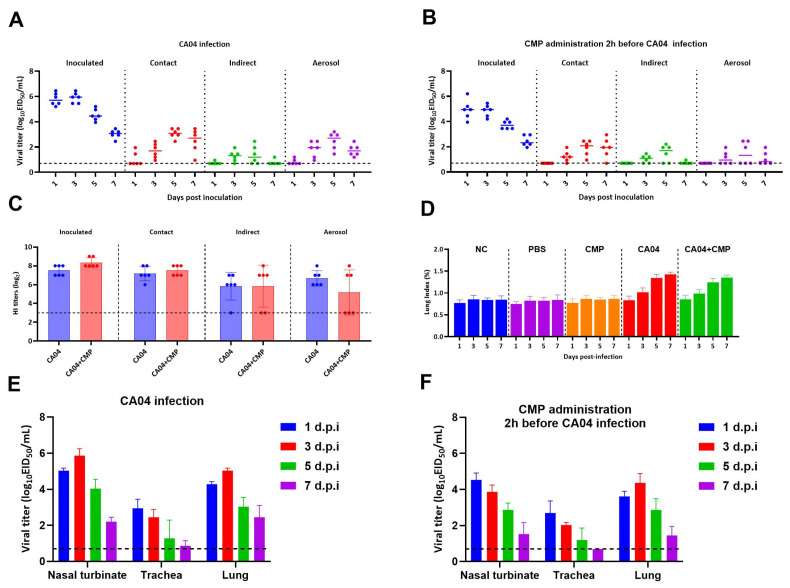
Guinea pig transmission efficiency and viral tissue distribution: (**A**,**B**) Nasal-wash viral titers of donors in CMP-pretreated versus untreated control groups; (**C**) Individual serum HI titers of guinea pigs in CMP-pretreated versus untreated control groups; (**D**) Lung index of guinea pigs; (**E**,**F**) Distribution of influenza virus in the respiratory tract (nasal turbinates, trachea, and lung).

In the CA04 infection group exposed to aerosol transmission, CA04 virus was detectable in nasal lavage fluid from all guinea pigs at 1, 3, 5, and 7 dpi (Figure 2A). In contrast, no viral titers were detected in nasal lavage fluid from guinea pigs in the CMP-administered group exposed to aerosol transmission at 1 dpi, and viral titers were detected only in nasal lavage fluid from 3 guinea pigs (Figure 2B). CMP reduced the aerosol transmission rate of CA04 from 100% (6/6) to 50% (3/6). This indicates that CMP can decrease the aerosol transmission efficiency of influenza viruses.

### 3.4. CMP Lowers Seroconversion in the Aerosol-Exposed Group

In the aerosol-exposed cohort, antibodies were detected in the serum of all guinea pigs in the CA04 infection group, whereas antibodies were detected in the serum of only 3 guinea pigs in the CMP-administered group, CMP reduced the aerosol transmission rate of CA04 from 100% (6/6) to 50% (3/6) (Figure 2C), consistent with the reduced nasal-wash titers noted above.

### 3.5. CMP Decreases the Lung Index in Infected Guinea Pigs

Lung index was measured at 1, 3, 5, and 7 dpi. The CA04 infection group showed significantly elevated lung index at 3, 5, and 7 dpi versus negative controls (Figure 2D). Pre-exposure administration of CMP significantly lowered the lung index relative to the infection group, indicating in vivo protection.

### 3.6. CMP Decreases Viral Replication in the Nasal Turbinates

In the respiratory tracts of infected guinea pigs, viral titers in the nasal turbinates of the CA04 infection group showed an increasing trend from 1 to 3 dpi, followed by a decreasing trend from 5 to 7 dpi (Figure 2E). In contrast, viral titers in the CMP-administered group exhibited a consistent downward trend and remained consistently lower than those in the CA04 infection group (Figure 2F). This suggests that CMP may suppress viral replication in the nasal turbinates.

### 3.7. Effects of CMP on Cytokines in the Lungs of Infected Mice

**Figure 3:**
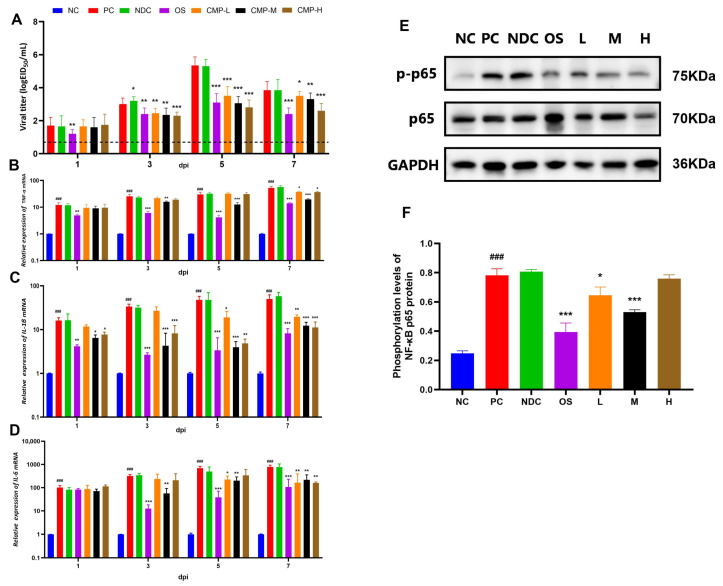
The immunomodulatory effects of CMP in vivo: (**A**) Viral titer in lung tissue. The mRNA levels of inflammatory cytokines TNF-α (**B**), IL-1β (**C**) and IL-6 (**D**) in lung tissue were detected using RT-qPCR. (**E**) Western blot electrophoresis bands of NF-κB p65 and p-NF-κB p65 in lung tissue from 5 dpi mice. NC: NC group; PC: PC group; OS: OS group; L: CMP-L group; M: CMP-M group; H: CMP-H group. Same legend applies below. (**F**) Phosphorylation levels of NF-κB p65 protein in mouse lung tissue post-infection (pp65/p65). ^###^ *p* < 0.001, vs. the NC group; * *p* < 0.05, ** *p* < 0.01, *** *p* < 0.001, vs. the PC group.

As shown in Figure 3A, at 3 dpi, the viral titer in lung tissue of the PC group was significantly higher than that in the CMP-H group (*p* < 0.001). Compared with the PC group, viral titers were reduced to varying degrees in all three treatment groups (CMP-L, CMP-M, CMP-H). At 1, 3, 5, and 7 dpi, expression levels of TNF-α (B), IL-1β (C), and IL-6 (D) in lung tissue were significantly higher in PC group mice than in the NC group (*p* < 0.001). No significant differences were observed between the NDC group and PC group. Compared with the PC group, all four treatment groups (OS, CMP-L, CMP-M, and CMP-H) exhibited varying degrees of suppression in TNF-α, IL-1β, and IL-6 expression. Cytokine expression levels significantly increased with infection duration, with oseltamivir and CMP-M exerting the most pronounced effects on cytokine levels.

### 3.8. The Regulatory Role of CMP in the NF-κB Signaling Pathway

As shown in Figure 3E,F, compared with the NC group, the PC group exhibited significantly elevated NF-κB p65 protein phosphorylation levels (*p* < 0.001). Compared with the PC group, the NF-κB p65 protein phosphorylation levels were significantly reduced in both the OS group and the treatment groups (CMP-L and CMP-M groups) (*p* < 0.05, *p* < 0.001), with the most pronounced reduction observed in the OS group and CMP-M group. These findings suggest that the NF-κB signaling pathway may play a crucial role in the immune modulation of CMP hosts.

## 4. Discussion

The present study demonstrated that CMP significantly inhibited replication of the CA04 influenza A virus in vitro and in vivo and partially blocked airborne transmission in a guinea pig model. These findings position CMP as a natural, low-toxicity antiviral candidate for prevention and control of influenza infection and spread, complementing current vaccine and drug-based strategies [17,52]. The transmission-blocking signal in our model aligns with the central role of airborne spread in influenza epidemiology and pathogenesis [1,53].

Aerosol interruption is particularly consequential because airborne spread is a major driver of IAV dissemination across hosts and settings [54]. Fine respiratory particles (<5 µm) can remain suspended for prolonged periods, traverse short to intermediate distances, and deposit deeply in the lower respiratory tract, thereby increasing infection probability and disease severity [55]. Environmental conditions—especially temperature and relative humidity—modulate virion stability and transmissibility, further amplifying outbreak risk in seasonally favorable climates and enclosed environments [56]. Airborne IAV and other respiratory viruses have been directly detected in exhaled breath and high-risk venues such as live-poultry markets, linking bioaerosol burden to human and animal exposure [27]. Ferret and guinea-pig models demonstrate that strictly airborne exposure suffices to initiate productive infection, underscoring aerosols as a self-sustaining transmission route independent of contact or fomites [57,58]. Treatment with Lentinan (LNT) significantly reduced viral shedding in the nasal cavity, decreased the transmission efficiency of SARS-CoV-2 aerosols, and potentially lowered the infectiousness of affected individuals [45]. Interventions that lower emitted or inhaled viral dose—whether by reducing upper-airway shedding or enhancing mucosal defenses—are therefore expected to curb onward spread [59]; our finding that CMP reduces nasal shedding and seroconversion in the aerosol-exposed cohort is consistent with this paradigm and suggests potential utility as a complementary, low-toxicity countermeasure at the human–animal interface.

**Figure 4:**
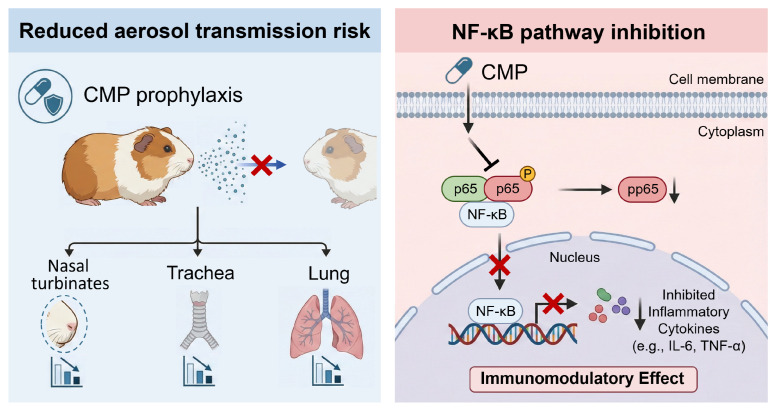
Proposed mechanism of CMP in mitigating influenza aerosol transmission and airway inflammation.

Polysaccharides, as a class of important biomacromolecules, exhibit remarkable “multi-target” activity in the field of biomedicine. Extensive scientific research confirms that polysaccharides not only directly inhibit various viruses but also broadly modulate the immune system, exerting clear protective effects in infection or injury models. Houttuynia cordata polysaccharides enhance survival rates in H1N1-infected mice, mitigate lung and intestinal damage, reduce viral replication, suppress inflammatory responses, protect the intestinal barrier, and regulate mucosal immunity [60]. The antiviral effects of Isatis root polysaccharides are closely linked to their inhibition of TLR-3 signaling pathway activation, thereby mitigating the upregulation of pro-inflammatory factors induced by influenza viruses [61]. Astragalus polysaccharides primarily function by blocking viral adsorption. They also activate macrophages, enhancing phagocytosis, and induce the production of multiple cytokines including interleukin-1 (IL-1) and tumor necrosis factor-alpha (TNF-α). This significantly improves clinical symptoms and lung damage in mice infected with H1N1 virus [62]. Schisandrin polysaccharides have been shown to interfere with the nuclear export process of viral nucleoproteins, thereby inhibiting viral replication [63]. This study revealed that beyond direct antiviral effects, CMP significantly suppressed H1N1 virus-induced p65 phosphorylation. This suggests that CMP may mitigate virus-induced inflammatory damage not only by directly inhibiting viral replication but also by intervening in the excessive activation of the NF-κB signaling pathway. (Figure 4). This provides potential molecular evidence for its “multi-target” antiviral and immunomodulatory effects, addressing the limitations of single-target antiviral drugs [46,47,48]. This characteristic not only broadens the spectrum of action for Chinese herbal polysaccharides but also lays the foundation for developing novel natural antiviral drugs. Such adjunctive strategies are particularly relevant given the historical emergence of resistance to neuraminidase inhibitors and the variable clinical performance of existing agents under field conditions [9,64]. By lowering viral load and reducing emission or susceptibility at the host level [65], CMP may contribute to reduced transmission efficiency, echoing evidence that interventions diminishing exhaled virus can curb spread [66,67,68].

This study suggests that CMP modulates the NF-κB pathway in an influenza virus infection model. However, whether this regulatory effect is specific to virus-induced inflammation or inherent to CMP’s broad-spectrum anti-inflammatory properties remains to be elucidated [69]. Future research should compare CMP’s impact on NF-κB and related inflammatory pathways under various classical pro-inflammatory stimuli (e.g., LPS, TNF-α) to clarify its spectrum of action and potential targets [70,71]. This work is crucial for precisely defining CMP’s clinical application scenarios, such as its specialized use for viral pneumonia or as a broad-spectrum anti-inflammatory agent. The study found that CMP’s in vitro cytotoxic concentration (CC_50_) is significantly higher than its effective in vivo dose. This discrepancy stems from the fundamental differences between static high exposure in vitro and dynamic absorption, metabolism, and immune regulation in vivo [72]. In vivo experiments revealed that CMP’s inhibition of NF-κB p65 phosphorylation did not exhibit strict linear dose dependency across tested concentrations (Figure 3F). This likely reflects its complex properties as a natural polysaccharide immunomodulator—balancing immune network regulation across different doses rather than exhibiting simple linear suppression or toxicity [73]. Pharmacodynamically, effect saturation may exist, with the medium dose (CMP-M) approaching maximum inhibitory efficacy. Additionally, as a crude macromolecular polysaccharide extract, its oral absorption and tissue distribution may exhibit pharmacokinetic saturation, limiting linear increases in target site concentrations at higher doses. Finally, complex interactions among CMP’s multiple components may contribute to non-linear synergistic effects [74]. Future studies should elucidate the mechanism underlying this nonlinear relationship through component purification and determine its optimal therapeutic window. The currently low selectivity indices (EC_50_/CC_50_) clearly indicate the direction for future optimization: namely, enriching or purifying single or homogeneous polysaccharide components bearing antiviral activity from CMP through activity-directed stepwise separation (such as ion exchange chromatography and gel filtration chromatography) [75,76]. Expanded in vivo studies—covering toxicity, immunogenicity, durability of protection, and head-to-head comparisons with standard of care—are warranted to support clinical translation. Mechanistic transmission studies (e.g., staged time-of-addition, contact vs. aerosol partitions, and quantification of emitted virus) will be valuable to confirm mode of action within established influenza transmission frameworks [53,66,77]. Collectively, these investigations will clarify CMP’s potential as an effective and safe natural antiviral for influenza and possibly other respiratory viruses.

## 5. Conclusions

*Cnidium monnieri* polysaccharides (CMP) showed measurable anti-influenza activity, inhibiting CA04 replication in vitro within non-cytotoxic ranges. In a guinea pig model, pre-exposure CMP reduced nasal shedding and partially blocked airborne transmission, as reflected by decreased seroconversion in exposed animals and reduced tissue viral loads. CMP also attenuated infection-associated inflammatory responses, suppressing TNF-α/IL-1β/IL-6 expression and reducing NF-κB p65 phosphorylation in vivo. Collectively, these findings support CMP as a promising low-toxicity, multi-target candidate for influenza prevention and transmission mitigation, warranting further fractionation/characterization and mechanistic–translational studies.

## Data Availability

The data supporting the findings of this study are available from the corresponding authors upon reasonable request.

## Supplementary Materials

The following supporting information can be downloaded at: https://www.mdpi.com/article/10.3390/v18010086/s1, Table S1 Monosaccharide composition of *Cnidium monnieri* polysaccharides; Table S2 Molecular Weight Results of *Cnidium monnieri* polysaccharides; Figure S1 HPGPC Chromatogram of *Cnidium monnieri* polysaccharides; Table S3 Purity of *Cnidium monnieri* polysaccharides.

## Abbreviations

The following abbreviations are used in this manuscript:

IAV	Influenza A virus
CMP	*Cnidium monnieri* polysaccharides
HSV	Herpes simplex virus
HBV	Hepatitis B virus
HIV	Human immunodeficiency virus
DMEM	Dulbecco’s Modified Eagle Medium
FBS	Fetal Bovine Serum
CCK-8	Cell Counting Kit-8
CC50	Median cytotoxic concentration
EC50	Median effect concentration
EID50	Median infective dose
TCID50	Median tissue culture infectious dose
WHO	World Health Organization
SPF	Specific pathogen free
TCM	Traditional Chinese Medicines
HI	Hemagglutination inhibition
