# Supplementary material for: Cnidium monnieri Polysaccharides Exhibit Inhibitory Effect on Airborne Transmission of Influenza A Virus
Source: Viruses. 2026 Jan 8;18(1):86. doi: 10.3390/v18010086 (PMC12846621; doi:10.3390/v18010086)
Supplement: Supplementary file 1 [file viruses-18-00086-s001.zip › viruses-4084162-supplementary.pdf]

Supplementary Materials

Title: *Cnidium monnieri* Polysaccharides Exhibit Inhibitory Effect on Airborne Transmission of Influenza A Virus

Supplementary Table 1 Monosaccharide composition of *Cnidium monnieri* polysaccharides

Name	Peak area （nC*min）	Retention time （min）	Molar ratio	Content (μg/mg)
Gal	26.258	15.384	0.384	73.916
Glc	28.959	17.542	0.162	31.145
GalA	10.928	42.875	0.139	28.910
Ara	12.504	11.842	0.148	23.734
Man	5.52	21.825	0.055	10.605
Rha	2.693	10.834	0.039	6.792
Rib	4.655	27.917	0.029	4.669
Xyl	4.095	20.75	0.027	4.322
GlcA	1.35	45.3	0.009	1.831
GlcN	1.47	13.184	0.005	1.103
Fuc	0.283	5.159	0.004	0.742
GalN	0.121	10.192	0.000	0.075

Supplementary Table 2 Molecular Weight Results of *Cnidium monnieri* polysaccharides

RT(min)	lgMp	lgMw	lgMn	Mp	Mw	Mn	Peak area ratio%
31.242	5.5	5.5	5.4	290876	288175	280560	0.607
37.048	4.7	4.7	4.7	53970	53612	52686	3.67
45.665	3.6	3.6	3.6	4430	4418	4403	18.42
47.093	3.5	3.5	3.5	2927	2921	2918	13.011
49.522	3.2	3.2	3.2	1447	1446	1449	16.799
51.732	2.9	2.9	2.9	762	762	767	21.326
54.113	2.6	2.6	2.6	382	382	386	26.167

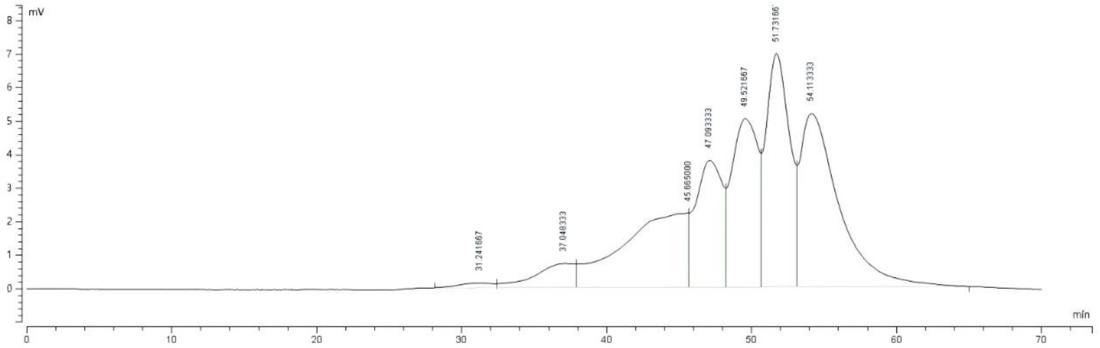

Supplementary Figure 1 HPGPC Chromatogram of *Cnidium monnieri* polysaccharides

Supplementary Table 3 Purity of *Cnidium monnieri* polysaccharides

Product Name	*Cnidium monnieri* polysaccharides	
Test	Specification	Results
Characteristics	Brown-orange powder	Complies
Purity(UV)	>90%	93.60%
